# Synthetic Cell‐Based Artificial Stem Cell Niches for Hematopoietic Stem Cell Differentiation

**DOI:** 10.1002/cbic.202500864

**Published:** 2026-04-21

**Authors:** Ivaylo Balabanov, Sara Madureira, Anna Burgstaller, Maja Fehlberg, Nils Piernitzki, Nurzhan Abdukarimov, Franziska Lautenschläger, Oskar Staufer

**Affiliations:** ^1^ INM ‐ Leibniz Institute for New Materials Saarbrücken Germany; ^2^ Helmholtz Institute for Pharmaceutical Research Saarland Helmholtz Center for Infection Research Saarbrücken Germany; ^3^ Center for Biophysics Saarland University Saarbrücken Germany; ^4^ Max Planck School Matter to Life Heidelberg Germany; ^5^ NT Faculty Experimental Physics Saarland University Saarbrücken Germany; ^6^ Max Planck Bristol Centre for Minimal Biology Bristol UK

**Keywords:** artificial cells, confinement, microwells, synthetic microenvironments, synthetic tissue

## Abstract

Hematopoietic stem cells (HSCs) receive a combination of biochemical and biomechanical signals within the bone marrow that guide their differentiation process. These include soluble factor signaling with cytokines, cellular confinement in the stem cell niche, and contact‐dependent receptor–ligand interactions with stromal cells. Recreating this complex microenvironment in vitro is a principal engineering challenge for regenerative therapies and tissue engineering. While cytokines can be easily supplemented in vitro, and several systems for confined HSC culture have been developed, integrating receptor‐based intercellular interactions found in stem cell niches has only been achieved with quantitatively undefined heterotypic co‐cultures. We report here the development of microwell‐based systems that integrate synthetic cells to mimic receptor–ligand interactions within hematopoietic niches. The synthetic cells are based on droplet‐supported lipid bilayers (dsLBs) with cytomimetic stiffness and present Notch receptor ligands on a laterally mobile lipid membrane. We show the system's applicability to individually tune the three signaling axes: soluble factors, confinement, and intercellular interactions for HSC differentiation. Introducing synthetic cells as an alternative to coculture and feeder cells opens the possibility to engineer precisely defined HSC niches with adjustable biochemical and biomechanical properties.

## 1 Introduction

Hematopoietic stem cell differentiation is a complex and highly regulated process, taking place in the bone marrow, that generates all blood and immune cell types, including erythrocytes, leukocytes, platelets, granulocytes, and dendritic cells (DCs) [1, 2, 3]. It involves a series of lineage commitment steps, governed by both intrinsic genetic programs and extrinsic cues from the cellular microenvironment.

Understanding and reproducing HSC differentiation in vitro benefit both basic research and the development of therapeutics, namely for hematologic malignancies and immunodeficiencies. Various in vitro models, generated over the years, have provided insight into the distinct molecular mechanisms governing both self‐renewal and lineage commitment. Many of them have focused on the transient use of extrinsic regulators like cytokine and growth factor cocktails, due to the simplicity and reproducibility of cultures [4, 5, 6, 7]. These include stem cell factor (SCF), Flt3 ligand (FLT3L), thrombopoietin (TPO), granulocyte‐macrophage colony‐stimulating factor (GM‐CSF), and various interleukins (IL) [3, 7, 8, 9, 10]. Other strategies employ co‐culturing of HSCs with supportive stromal cells, mimicking the bone marrow niche and enhancing differentiation efficiency and lineage fidelity [4, 11].

It has now been recognized that in addition to cytokines, multiple other factors cooperate in determining the fate of HSCs *in vivo*. The bone marrow microenvironment presents unique biochemical and biophysical characteristics, such as distinct pH, confinement, elasticity, and shear stress, which collectively contribute to HSC self‐renewal, quiescence, and lineage commitment through various mechanisms [12]. Microenvironmental pH levels affect metabolic pathways like polyamine synthesis [13]. Moreover, HSCs can sense and respond to matrix stiffness and fluid flow using mechanosensitive ion channels, primary cilia and integrins [14]. Cumulatively, these factors affect the homing, engraftment, and retention of HSCs within osteogenic and perivascular niches [15]. Recent studies using microfabricated and soft hydrogel platforms have demonstrated how spatial limitations will accelerate the depletion and differentiation of HSCs while loss of topographical cues can accelerate HSC exhaustion or differentiation [16, 17]. These results show that multiple characteristics of HSCs and the bone marrow microenvironment can be exploited with engineered synthetic materials to enable precise in vitro modulation of HSC fate [18].

Microfabricated nanowells and microwells have emerged as powerful tools for precisely confining single cells and controlling their microenvironment. These structures enable spatial restriction that affects cell shape, cytoskeletal organization, and function without compromising viability, which is critical for studying mechanobiology at the single‐cell level [19]. Nanowell platforms have also been adapted for single‐cell secretion analysis, allowing high‐throughput measurement of functional heterogeneity and enabling applications in drug screening and antibody discovery [20]. More recently, combined nanowell‐in‐microwell arrays facilitate automated imaging and tracking of thousands of individual cells, supporting advanced morphological profiling and machine learning–based phenotype classification [21]. These devices represent important advances in mimicking physiological confinement and enabling high‐resolution analysis of cell behavior.

HSC confinement does not only facilitate cytokine signaling, but also boosts contact‐dependent interaction between HSCs and stromal cells. One of the critical contact‐dependent intercellular interaction axes that modulate stem cell fate is the Notch pathway. In the embryonic setting, Notch signaling is required for the generation of definitive HSCs, while in adult hematopoiesis, its effects are context‐dependent and tightly regulated [22]. Activation of Notch receptors in HSCs can preserve stemness and prevent premature differentiation, but excessive or prolonged Notch activation can block it or cause lineage skewing [23]. Notch also interacts with other pathways such as Wnt, mTOR, or JAK/STAT [24, 25] and contributes to the adaptation of HSCs within their specific niches, such as by modulating adhesive interactions and supporting quiescence; however, cell‐to‐cell heterogeneity and niche‐derived cues significantly shape Notch signaling outcomes [26]. Overall, accurately controlled Notch activity is essential for proper HSC function, and its dysregulation is implicated in hematological diseases and in altered regeneration capacity [22]. Nevertheless, the precise spatiotemporal presentation of Notch ligands in vitro has remained a challenge. Stromal cocultures provide limited control over ligand density and distribution, making it difficult to dissect quantitative signaling inputs. Thus, technologies that allow for the defined and tunable display of Notch ligands in a physiologically biomimetic context are critically needed to unravel the multifactorial regulation of HSC fate ex vivo.

Synthetic cell engineering has emerged as a powerful framework to functionally mimic cellular microenvironments with increasing precision. Among its most promising tools are synthetic cell models, constructed from engineered lipid membranes, which are capable of recapitulating key biochemical and biomechanical features of living cells [27]. These artificial cells enable the controlled presentation of defined quantities of receptor ligands, exhibit membrane fluidity, and display tunable mechanical properties. Such synthetic cell systems are progressively closing the gap between artificial constructs and natural cellular systems, offering new opportunities to study contact‐dependent intercellular signaling in well‐defined yet biomimetic contexts [28].

In this study, we aim to integrate synthetic cells into a modular ex vivo culture platform that allows us to independently tune and study three key cues for HSC differentiation: soluble cytokine signaling, mechanical confinement, and receptor‐mediated interactions with stromal cells. By combining defined cytokine signaling, spatial confinement, and synthetic cell‐mediated presentation of stromal ligands, we establish a minimal yet multifunctional system that enables the systematic modulation of central biochemical and biophysical cues in HSC differentiation. This synthetic cell engineering approach enables the bottom‐up reconstruction of a functional hematopoietic stem cell niche under fully defined and tunable conditions.

## 2 Results

To reconstruct the HSC niche in vitro, we established a modular culture platform integrating key biochemical, biomechanical, and cellular features of the bone marrow microenvironment. We focused on three core components of HSC regulation: soluble cytokine signaling, mechanical confinement, and receptor‐mediated interactions with stromal cells.

### 2.1 Cytokine‐Driven Differentiation of Hematopoietic Progenitors

**FIGURE 1:**
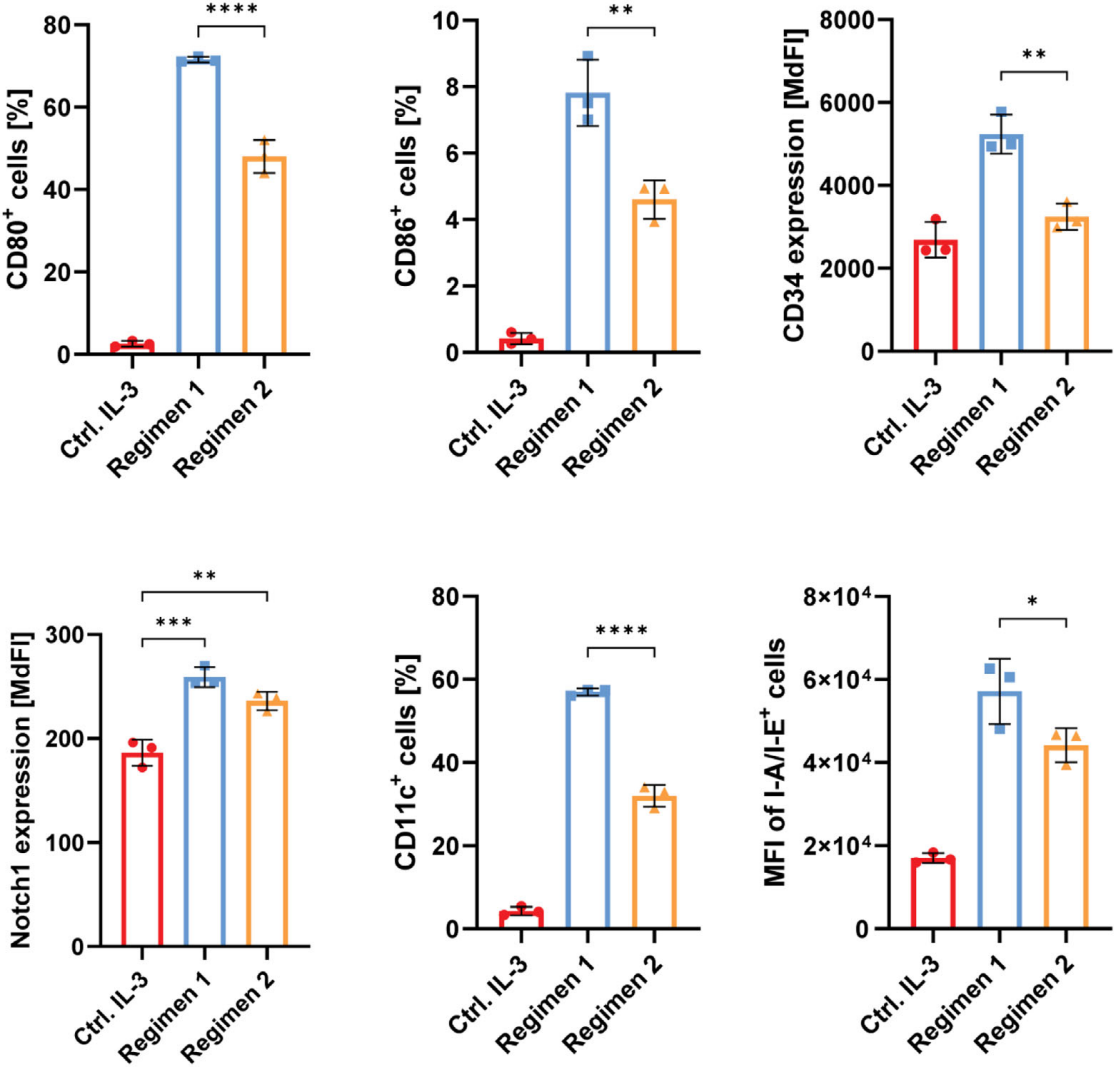
HSC differentiation to DCs in 2D. Flow cytometric analysis of FDCP‐Mix cells after 10‐day differentiation in 2D culture, induced by administration of GM‐CSF and IL‐4 in two distinct regimens. For control, cells were maintained in IL‐3‐supplemented medium as a necessary survival signal. Data is represented as either positive population of singlets, median fluorescence intensity (MdFI) of singlets, or mean fluorescence intensity (MFI) of positive population. Results are shown as mean ± SD from *n* = 3 technical replicates from one of two independent experiments. Statistical analysis was performed with one‐way ANOVA, combined with Tukey's multiple comparisons test, where *p < 0.05; **p < 0.01; ***p < 0.001; ****p < 0.0001; and ns is nonsignificant.

As a biochemical foundation, we applied a minimal set of soluble factors known to direct monocyte‐derived dendritic cell lineage commitment in HSCs, adopting as a model the murine hematopoietic progenitor cell line FDCP‐Mix [4]. In the absence of supporting stroma, these cells can be maintained in vitro under standard 2D suspension culture conditions by continuous supplementation of the self‐renewal factor IL‐3. Based on a previous study in which FDCP‐Mix cells were differentiated into DCs over 16 days [29], we designed and tested two regimens for differentiation over a reduced 10‐day period. In both, GM‐CSF was provided regularly from the onset to drive myeloid commitment and progression through granulocyte–macrophage progenitors toward monocytes. In the first regimen, IL‐4 was introduced periodically from day four to direct monocyte‐to‐DC conversion and maturation, while in the second, IL‐4 was added in a single dosage at day eight. Quantification of the expression of surface markers by flow cytometry revealed robust increase of canonical differentiation markers, including clusters of differentiation CD80, CD86, CD11c, and class II major histocompatibility complex (MHC‐II, in the figures represented as I‐A/I‐E, corresponding to the allelic variants, expressed by the cell line) molecules, demonstrating progress along the common myeloid progenitor pathway (Figures 1 and S1). Specifically, for CD80 and CD86, key costimulatory molecules for antigen‐presenting cells, we observed prominent upregulation in the cultures. This was also the case for CD11c, a classical DC marker. Interestingly however, expression of the stem cell marker CD34 was higher after the 10‐day treatment, suggesting an active progenitor expansion and partial lineage commitment, rather than terminal differentiation to DCs [30]. This concept was supported also by the increased presence of Notch1 receptors on the cell surface, indicating a higher susceptibility of these precursors for Notch ligands. Most importantly, we could show an effective time‐reduced differentiation protocol, where earlier exposure to IL‐4 induced a stronger phenotypic shift, both in population‐wide expression frequencies and in fluorescence intensities, compared with the original treatment scheme. In summary, these results highlight the eminent role of biochemical signaling for initiation of hematopoietic differentiation.

### 2.2 Spatial Confinement and Mechanical Environment Modulate Cytokine Responsiveness

**FIGURE 2:**
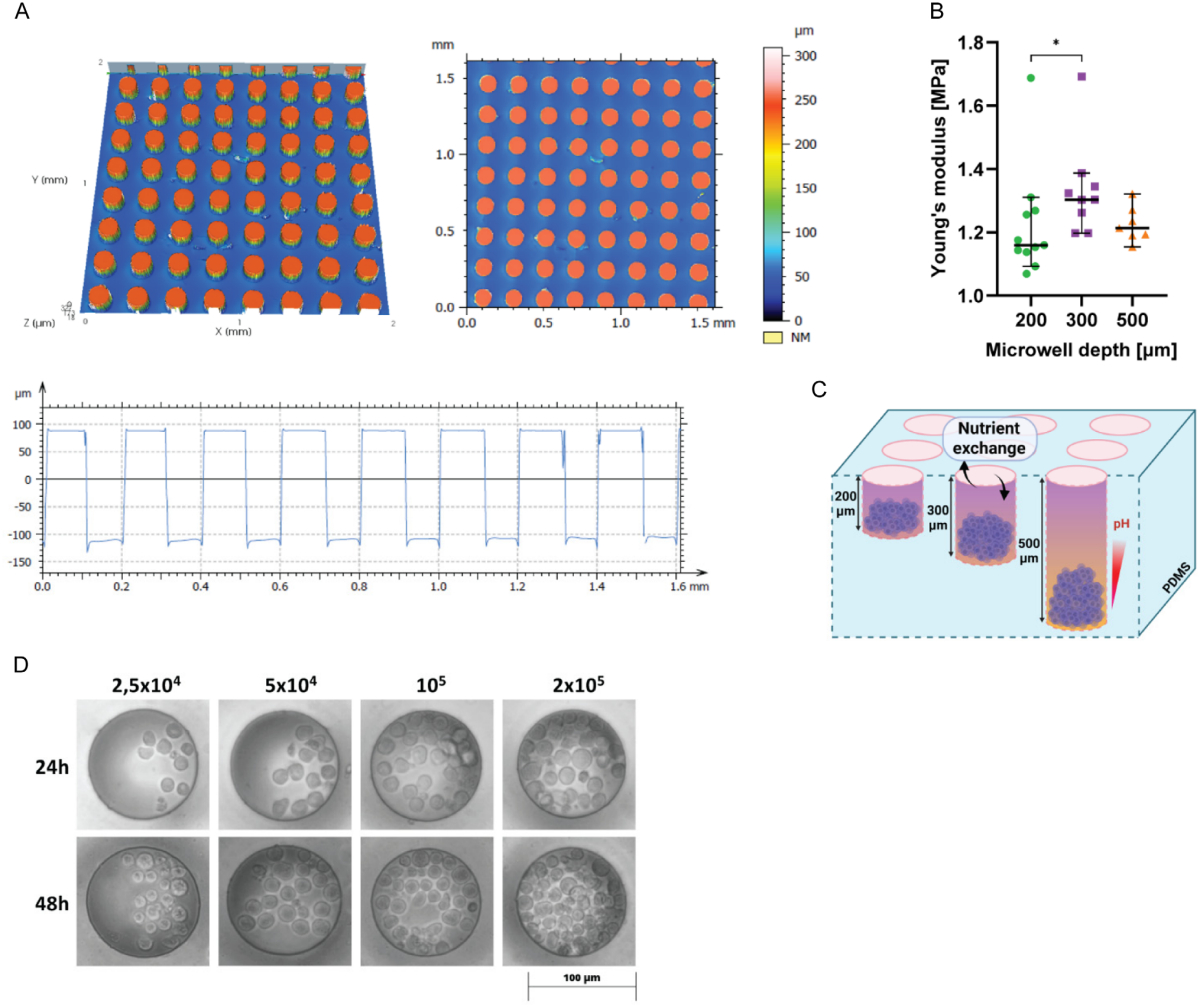
Characterization of microwell‐based 3D microenvironment for HSC culturing and differentiation. (A) Confocal microscopy‐based topographical analysis of a silicon wafer, used as a mold for the generation of microwell‐containing PDMS substrates. The plot below shows height profile derived from the 3D reconstructions. (B) Nanoindentation analysis showing comparison of the surface stiffness of all three studied PDMS substrate samples. Data from 10 independent measurements of each sample, shown as median + 95% CI from one out of two independent experiments. Statistical analysis was carried out with Kruskal–Wallis test, combined with Dunn's multiple comparisons test, where *p < 0.05; **p < 0.01; ***p < 0.001; ****p < 0.0001; and ns is nonsignificant. (C) Schematic representation of microwells of increasing depth, seeded with cells. The color gradient indicates impaired nutrient exchange in the deeper wells, leading to a decrease in pH. Samples of each microwell depth were produced separately. (D) Representative light microscopy images of FDCP‐Mix cells of different density growing over time inside microwells.

To incorporate the biomechanical constraints of the hematopoietic niche into our culture system, we engineered a polydimethylsiloxane (PDMS)‐based microwell platform with precisely tunable geometries. This approach provides three key advantages over conventional 3D culture systems: (1) microwell dimensions can be fabricated with high fidelity and reproducibility using photolithographically patterned silicon wafer templates (Figure 2A); (2) substrate stiffness can be modulated by adjusting the cross‐linking ratio of PDMS, enabling mechanical environments and microanatomical architecture that approximate those found in trabecular bone [31] (Figure 2B) (stroma mechanics are mimicked by synthetic cells as described below); and (3) unlike encapsulating hydrogels or droplet‐based platforms, microwell architecture allows differential control of nutrient and metabolite diffusion from the bulk culture medium to the cells by varying well depth (Figure 2C).

Different culturing substrates with a uniform pattern of microwells with a lateral diameter of 100 µm and varying depths of 200, 300, or 500 µm were fabricated by PDMS molding technique with microstructured silicon wafers as master structures. These wafers were processed by SF_6_‐based deep reactive ion etching using a photolithographically defined mask. Using higher base‐to‐curing agent ratio of 12:1 (w/w) (compared to standard 10:1 formulations), we obtained a substrate with higher compliance, which supported HSC growth and viability to an identical level as standard polystyrene cell culture plates (Figure S3A). The height profile of the wells was characterized by confocal microscopy (Figure 2A). To evaluate their capacity to promote spatial confinement for HSC cells, FDCP‐Mix hematopoietic progenitor cells were seeded onto the microwell substrates at varying densities (Figure 2D). Microscopic imaging immediately after seeding revealed a random distribution of cells across the surface, including both microwell interiors and inter‐well regions. Over a 24‐h period, however, cell migration and gravitational settling led to the vast majority of cells relocating into individual microwells and forming spatially confined multicellular clusters. This spontaneous confinement, driven by passive motility and local well geometry, recapitulates the topographical constraints experienced by HSCs *in vivo*. Next, we assessed the mechanical properties of the PDMS, which are relevant to cellular mechanosensing during interaction with the substrate, i.e., the most upper layer of PDMS in between the microwells, by performing nanoindentation analysis on the PDMS surface (Figure S2). We found the Young's modulus of this PDMS layer to be in the lower megapascal (MPa) range, closely resembling that of the trabecular bone to which HSCs make direct contact *in vivo* [31, 32, 33] (Figure 2B). The apparent differences between samples of different depth are likely attributable to surface defects originating from the silicon wafer, to inhomogeneous mixing, as well as to the coating procedure required for PDMS detachment, whose chemical interaction can locally inhibit or perturb the curing process [34].

**FIGURE 3:**
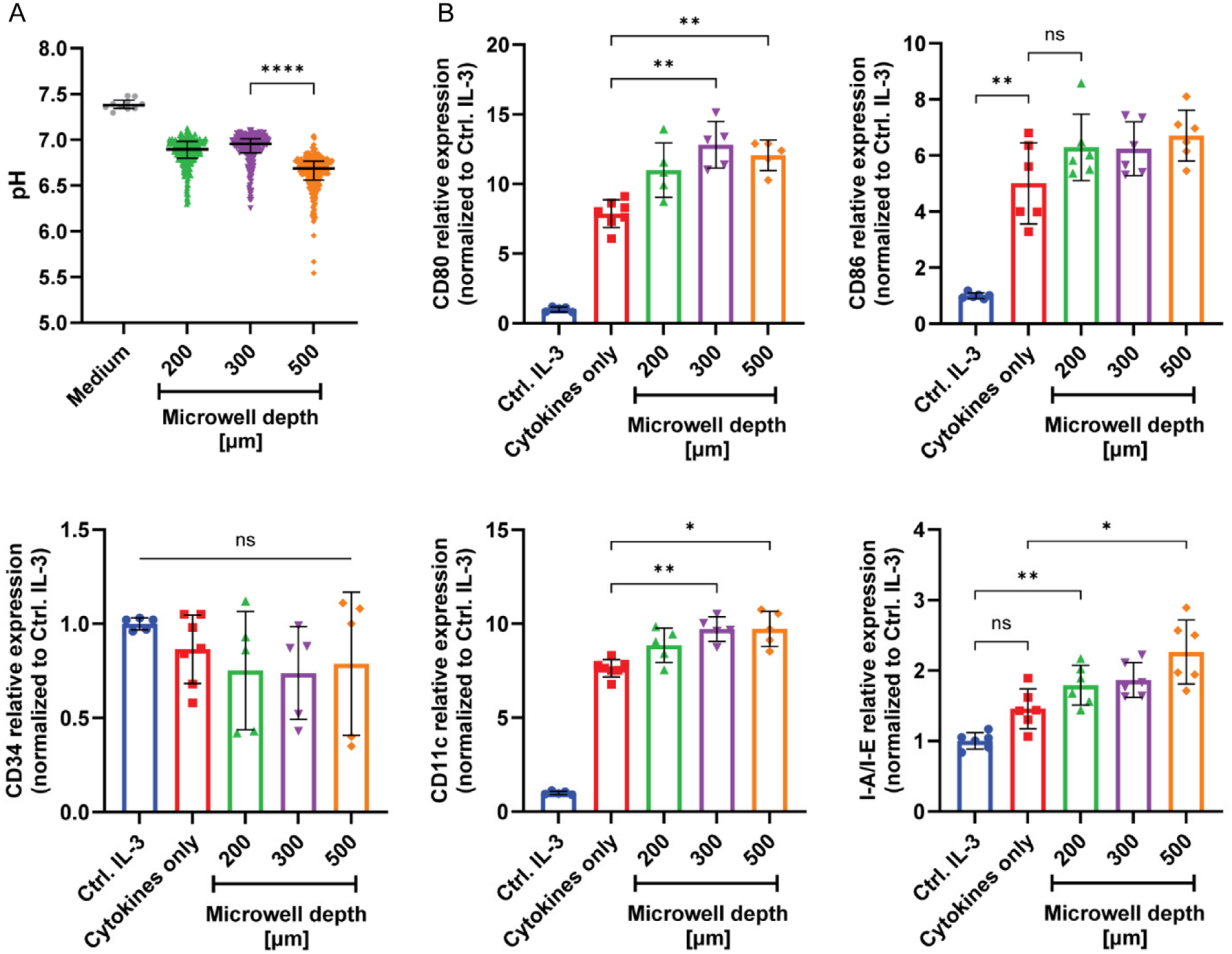
Effects of 3D microenvironment on HSC differentiation in microwells. (A) Fluorescence‐based ratiometric pH‐sensing measurements, performed in microwells of different depth with FDCP‐Mix cells, cultured for 72 h, indicating a progressive acidification of cellular microenvironment with the increase of microwell depth. Combined data from > 100 individual microwells per sample, shown as median + interquartile range from one out of two independent experiments. Statistical analysis was carried out with Kruskal–Wallis test, combined with Dunn's multiple comparisons test, where *p < 0.05; **p < 0.01; ***p < 0.001; ****p < 0.0001; and ns is nonsignificant. (B) Flow cytometric analysis of FDCP‐Mix cells after a 10‐day differentiation in 2D culture (red) versus in microwells of different depth. In both conditions, the HSCs were treated with the same regimen of GM‐CSF and IL‐4. For control, cells were maintained in IL‐3‐supplemented medium as a necessary survival signal. Data were normalized against the IL‐3 control group (blue), pooled from two out of three independent experiments and shown as mean ± SD from *n* = 6 technical replicates. Statistical analysis was performed with Brown–Forsythe and Welch ANOVA tests, combined with Dunnett's T3 multiple comparisons test, where *p < 0.05; **p < 0.01; ***p < 0.001; ****p < 0.0001; and ns is non‐significant.

Next, we aimed to assess whether the different microwell depths could modulate the degree of nutrient and waste product diffusion and thus lead to a progressive acidification of the microenvironment of the cell clusters at the bottom of the wells. For this, a fluorescence‐based ratiometric pH‐sensing method was implemented based on silica microspheres with a mean diameter of 4 µm, coated simultaneously with two fluorophores, a pH‐sensitive carboxyfluorescein (CF) and a pH‐insensitive cyanine 5 (Cy5) for signal normalization (see the Materials and Methods section). To ensure that measurements reflected extracellular pH in the microwells, particles were added onto the cultures 72 h after cell seeding and only 30 min ahead of measurement, minimizing the probability of internalization, despite the relatively large size of the particles. Fluorescence intensity analysis revealed a depth‐dependent acidification gradient, with 500‐µm‐deep wells reaching median pH values of 6.68 ± 0.2, while shallower wells of 200‐ and 300‐µm depth retained a higher pH of 6.9 ± 0.15 and 6.95 ± 0.17 respectively (Figure 3A). These results could not be traced down to discrepancies in cell proliferation inside the deepest microwells, as neither cell cycle nor viability showed any changes between the microwell cultures and cells grown in standard surface‐treated polystyrene plates (Figure S3B,D). Furthermore, pH of cultures, treated with differentiating cytokines for 8 days, was noticeably decreased in comparison to control cells, maintained with IL‐3, suggesting a larger role of environment acidification in HSC differentiation (Figure S3C). Together, these results support a confinement‐enhanced shift in metabolic exchange, mirroring hypoxic‐acidic zones observed in native HSC niches which show pH values between 6.7 and 7.5 [35]. Toward assessing if microwell confinement and the formation of acidic niches modulate lineage commitment, we cultured the HSC cells in the microwell format for a 10‐day period under the GM‐CSF + IL‐4 cytokine regimen 1, determined above. Differentiation was again assessed by flow cytometry. We found specific differential expression of several markers, namely CD80, CD86, CD11c, and MHC‐II with modest but consistent changes in expression in confined cultures cultured in microwells of different depths as compared to cultures differentiated in standard 2D culture wells (Figure 3B). Statistically significant differences between the generated well depths could be measured for all markers. However, for CD86, CD11c, and I‐A/I‐E, the deepest wells yielded the most pronounced effect as compared to the standard 2D well plate culture, indicating the transition from stem cell toward differentiated state was being affected. Taken together, while less dominant than cytokine signaling, the biomechanical and biophysical context in the form of confinement can modulate and potentially even boost lineage commitment, particularly in synergy with metabolic cues, such as pH shifts.

### 2.3 Synthetic Cells Induce Contact‐Dependent Signaling by Mimicking Stromal Interactions

**FIGURE 4:**
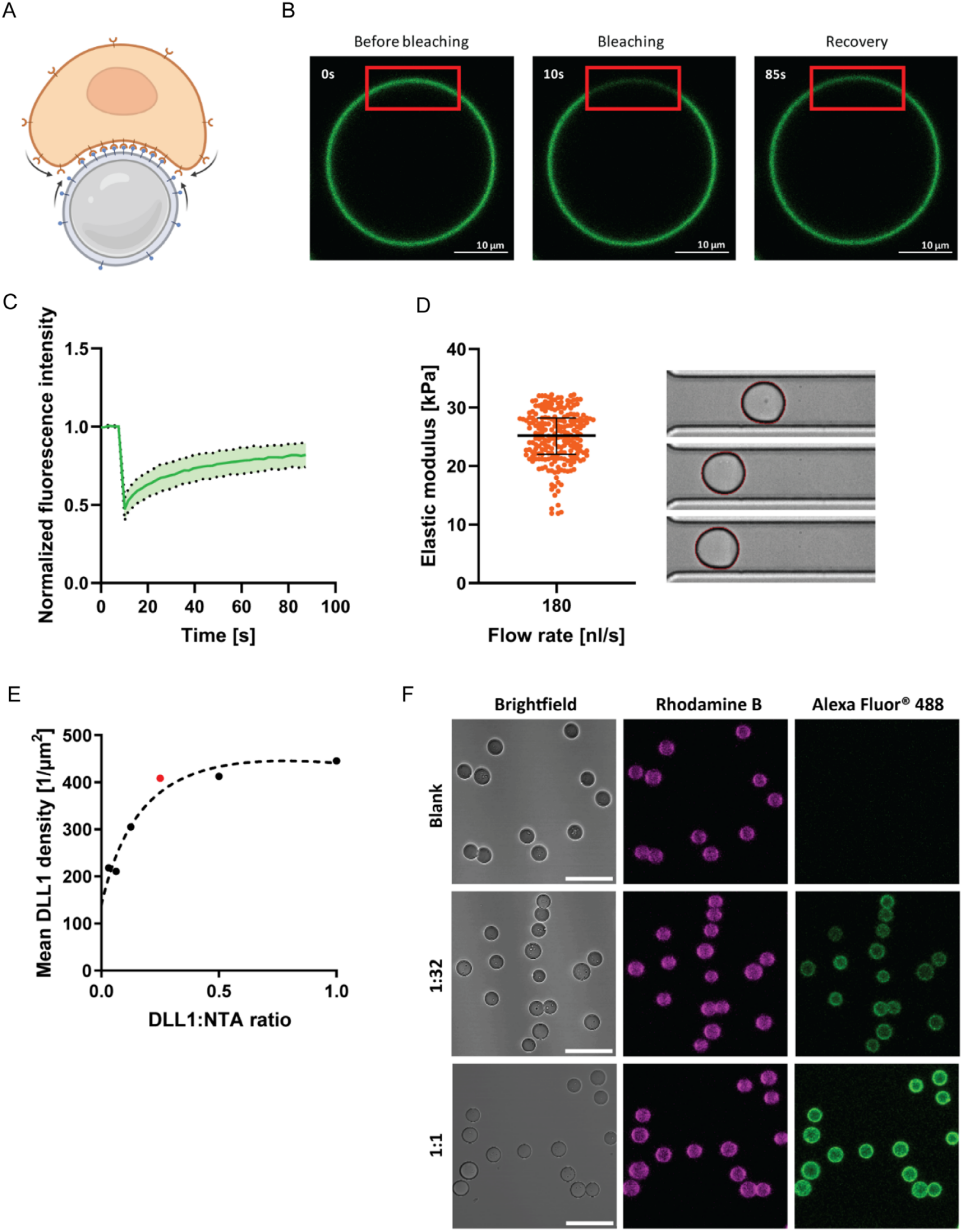
Characterization of biophysical properties of emulsification‐based droplet‐supported lipid bilayers (dsLBs). (A) Schematic representation of dsLB‐HSC cell contact. Receptor*–*ligand interactions are improved by the physiological stiffness and receptor mobility on both cell membranes. (B) Representative confocal microscopy images of an individual dsLB before, during, and after photobleaching, proving fluidity of the synthetic cell membrane. (C) Double normalized fluorescence intensity curve of FRAP experiments from (B). Line indicates mean of eight measurements in separate dsLBs; green area depicts the SD from these measurements. (D) RT‐DC measurements showing physiological elasticity of dsLBs under sheer stress. Each data point indicates a single dsLB and median ± interquartile range of the set is shown. On the right, snapshots of individual dsLBs deforming, while passing through the microfluidics channel with 20 µm diameter. (E) DLL1 binding curve, representing coupling of an array of protein‐to‐NTA ratios, generated from the MESF bead assay. The curve flattens at the ratio 1:4 (dot marked in red), corresponding to a density of ≈400 molecules per μm^2^ *—*the ratio used for all further assays with DLL1 dsLBs. (F) Representative confocal microscopy images, collected during the Bead assay from (E). Conditions shown include the lowest and highest DLL1:NTA ratios of 1:32 and 1:1, in addition to the blank without protein. Membrane rhodamine B is colored in magenta and DLL1‐AF488 in green. Scale bar corresponds to 50 μm.

As the third component of our bottom‐up culture system, we aimed to incorporate synthetic cells into the spatially confined hematopoietic progenitor clusters within microwells. These synthetic cells were designed to mimic key functional attributes of stromal cells in the native HSC niche, which not only secrete paracrine factors but also establish direct cell–cell contacts that drive receptor‐ligand signaling, involved in lineage commitment. To effectively substitute stromal inputs, the synthetic cells needed to fulfill two essential criteria (Figure 4A): (1) they must present ligands within a laterally mobile lipid bilayer to allow dynamic reorganization and receptor clustering at the contact site, and (2) they should exhibit mechanical properties within the range of natural stroma cells [36, 37], significantly softer than the rigid PDMS substrate, to support physiologically relevant biomechanical interactions. To meet these requirements, we employed droplet‐supported lipid bilayers (dsLBs). DsLBs are synthetic cells, based on oil‐in‐water emulsions with mean droplet size of 17.3 ± 2.5 µm (Figure S4) coated with a fluorescent lipid bilayer. dsLBs were formed using a silica membrane‐assisted emulsification strategy (see the Materials and Methods section). Membrane fluidity was verified by fluorescence recovery after photobleaching (FRAP) of Atto 488‐conjugated lipids within the lipid bilayer. FRAP analysis confirmed the lateral diffusion of membrane components with an average mobile fraction of 74 ± 18% and a t_1/2_ recovery time of 23.3 ± 14.4 s (Figure 4B,C). Next, the mechanical properties of individual dsLBs were assessed via real‐time deformability cytometry (RT‐DC), revealing an average Young's modulus of 25 ± 4.42 kPa, comparable to native stromal cells (e.g., osteoblasts) in the HSC niche in the kPa range [36, 37, 38] (Figure 4D).

**FIGURE 5:**
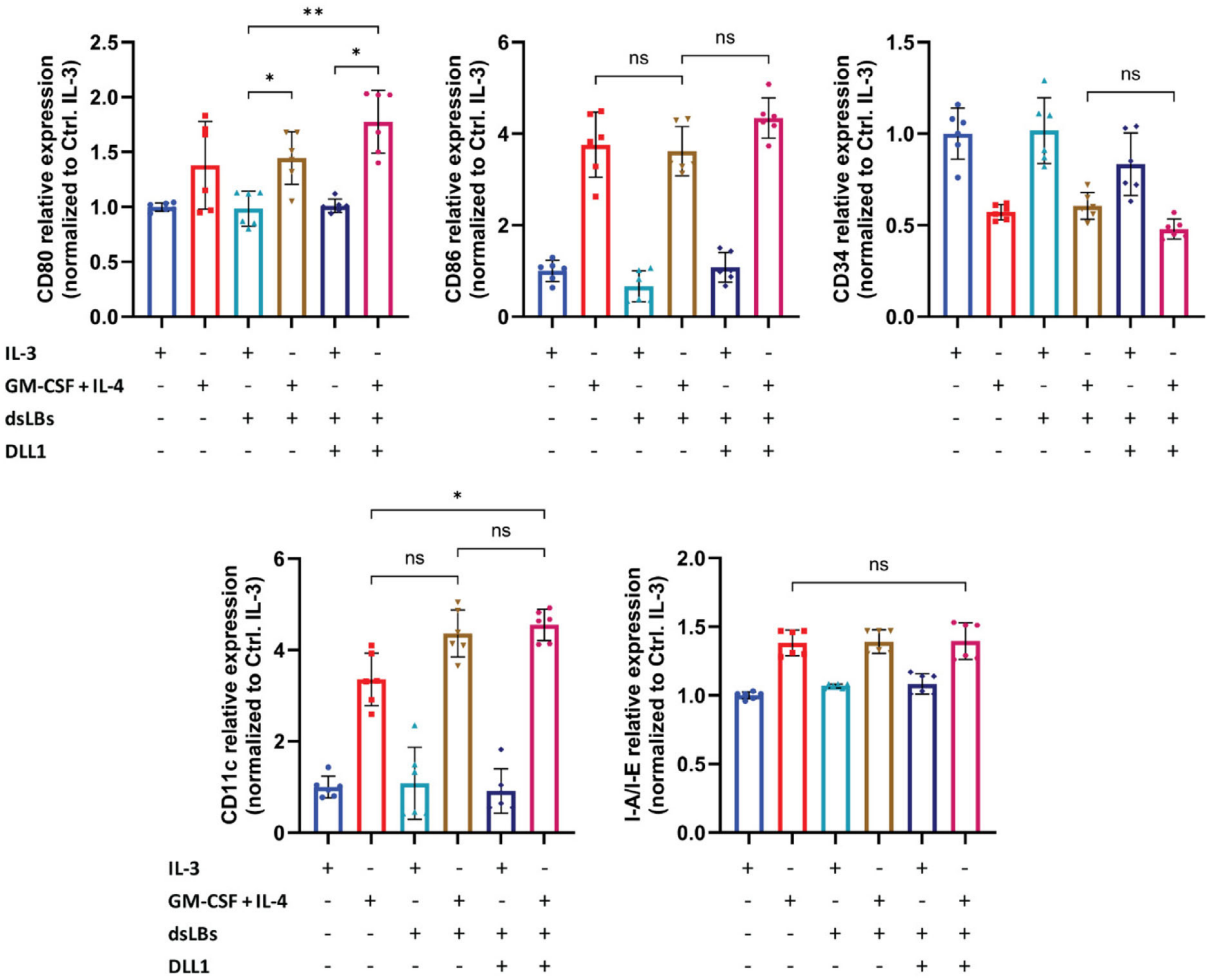
Effects of Notch activation on HSC differentiation by DLL1‐coated dsLBs in addition to cytokine administration in 2D culture. Flow cytometric analysis of FDCP‐Mix cells after 10‐day differentiation in half area 96‐well plate in the presence of GM‐CSF and IL‐4 alone, or in combination with either plain or DLL1‐functionalized dsLBs. All data is normalized to the control IL‐3‐treated group. Results are shown as mean ± SD from *n* = 6 technical replicates from two independent experiments. Statistical analysis was performed with Brown–Forsythe and Welch ANOVA tests, combined with Dunnett's T3 multiple comparisons test, where *p < 0.05; **p < 0.01; ***p < 0.001; ****p < 0.0001; and ns is nonsignificant.

To enable contact‐dependent signaling, we functionalized the lipid bilayer with mouse recombinant Delta‐like protein 1 (DLL1), a canonical Notch1 ligand involved in hematopoietic development. Ligand presentation was achieved through incorporation of NTA(Ni^2+^)‐functionalized lipids into the bilayer, allowing for site‐specific immobilization of C‐terminus *His*‐tagged DLL1. We utilized a DLL1 coupling density of 409 ± 58 molecules/µm^2^ on the dsLB surface, as determined by beads with known molecules of equivalent soluble fluorochrome (MESF) and visualized by confocal microscopy (Figure 4E,F). Such density falls in the same order of magnitude as strongly expressed native receptors and well within the range that has been explored in mechanistic studies [39]. Importantly, we have previously shown that under these coupling conditions, proteins are retained on the dsLB surface for over 48 h in a functional state and under cell culture media conditions [40]. Coculture of DLL1‐presenting dsLBs with FDCP‐Mix cells under 2D suspension conditions resulted in consistent, although nonsignificant trends in the expression of CD80, CD86, CD34, and CD11c in comparison to nonfunctionalized dsLBs or cytokine treatment alone, indicating augmented differentiation by the Notch signaling (Figure 5). Importantly, control dsLBs lacking DLL1 did not induce comparable changes in marker expression, nor did DLL1‐presenting dsLBs in the absence of cytokine stimulation elicit measurable changes in stem cell phenotype. These findings indicate that while cytokine signaling remains the dominant driver of lineage commitment, contact‐dependent Notch activation via synthetic cells can affect this process already under non‐confined conditions. Taken together, these results demonstrate that synthetic cells can functionally engage hematopoietic progenitors through productive receptor‐ligand interactions, offering a modular and tunable platform to reconstruct stromal signaling axes under fully defined conditions.

### 2.4 Full Integration of Biochemical, Biomechanical, and Synthetic Cellular Components Enhances Differentiation Outcomes

**FIGURE 6:**
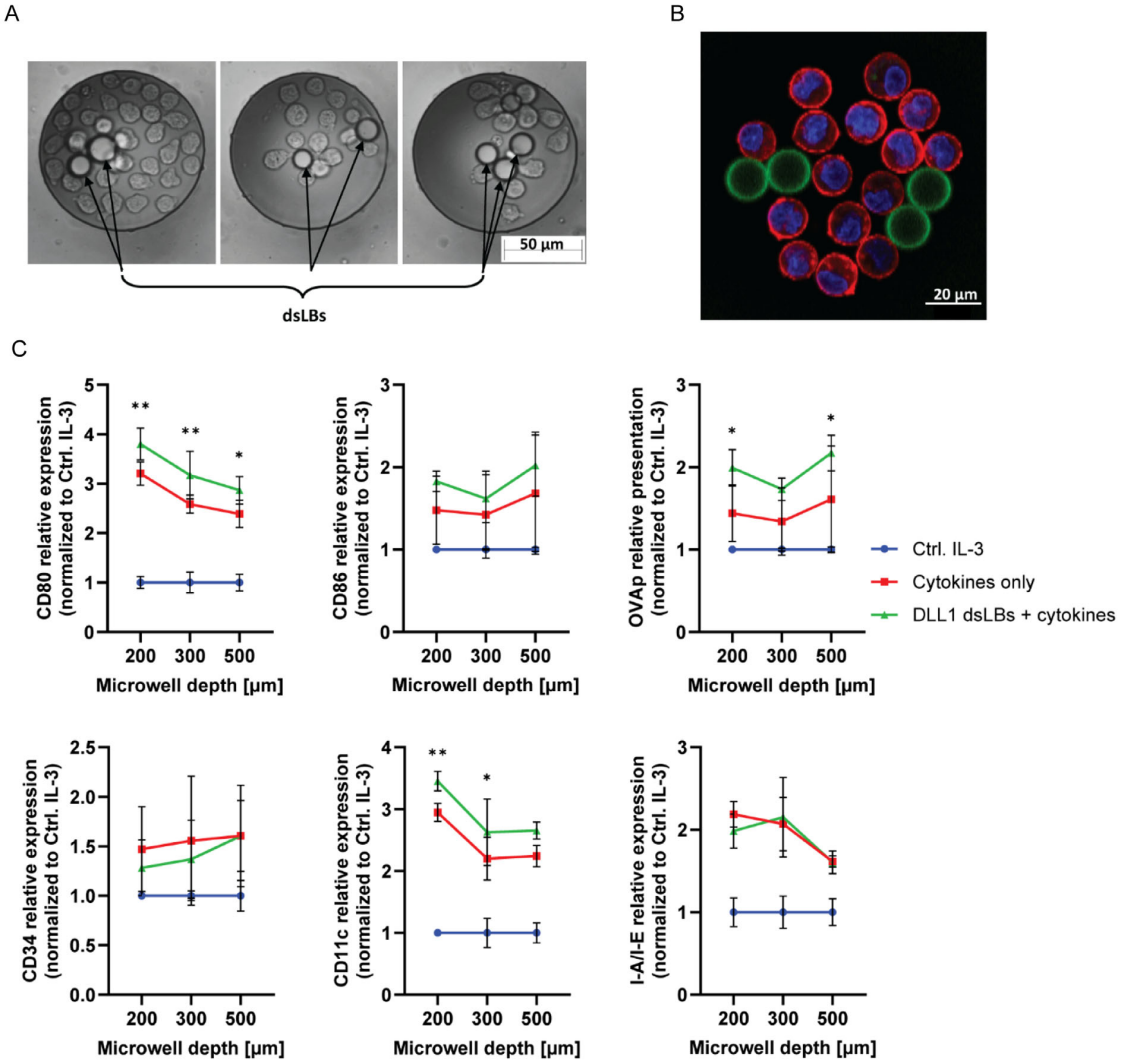
Integration of physical confinement, Notch, and cytokine signaling for HSC differentiation. Representative (A) light and (B) confocal microscopy images of FDCP‐Mix cells cultured with dsLBs inside microwells, showing continuous contact between the stem and synthetic cells. The HSCs are stained with phalloidin (red) and Hoechst (blue); dsLBs are shown in green. (C) Flow cytometric analysis of FDCP‐Mix cells after 10‐day differentiation inside microwells, triggered by either cytokines alone (red), or in combination with DLL1‐functionalized dsLBs (green), in comparison with controls, treated with IL‐3. Results show induction of DC markers, as well as antigen presentation capacity on the cell surface. The plots represent data from two independent experiments with three technical replicates each, normalized against the control IL‐3 group and depicted as mean ± SD. Statistical analysis was performed with two‐way ANOVA, combined with Tukey's multiple comparisons test with a single pooled variance. The statistical results, shown as *p < 0.05; **p < 0.01; ***p < 0.001; ****p < 0.0001; and as ns for non‐significant, display simple effects within rows comparisons between the DLL1 dsLB + cytokines (green) and the cytokines only (red) groups.

To assess potential synergy between the three components in the system, we combined cytokine stimulation, microwell confinement, and DLL1‐presenting synthetic cells. FDCP‐Mix cells were cultured in microwells of varying depths in the presence of GM‐CSF, IL‐4, and DLL1‐functionalized synthetic cells (Figure 6A,B). This integrated system yielded additive effects on dendritic cell differentiation markers. Conversion to DC precursor phenotype was evident in all microwell depths, with moderate increase of CD80, CD86, and CD11c expression over the cytokine‐only‐treated cells. Subsequent maturation showed signs of inhibition in parallel with increasing well depth, seen as a decrease in costimulatory CD80 receptor staining, as well as in MHC‐II expression, combined with an increase in CD34, specifically at 500‐µm depth (Figure 6C). In order to test the functionality of the differentiated cells for antigen presentation, they were loaded with fluorescently labeled peptide, derived from chicken ovalbumin (OVA). This OVA peptide 323–339 with the sequence ISQAVHAAHAEINEAGR is specific for the I‐A^*b*^ MHC‐II molecules, expressed by the FDCP‐Mix cell line. Subsequent flow cytometry measurements showed efficient binding of the OVAp upon differentiation with minor differences between conditions. **The OVAp presentation most effectivly increased for the samples cultured together with synthetic cells presenting DL‐L1.** These findings establish a functionally integrated ex vivo platform that recapitulates essential biochemical and biophysical features of the hematopoietic niche, allowing systematic dissection of individual and synergistic contributions of cytokines, physical constraints, and stromal cell mimicry.

## 3 Discussion

HSC differentiation is a tightly orchestrated process in which several biochemical and biomechanical cues contribute to the lineage commitment of HSCs. The system we present here combines several of these, but most importantly integrates synthetic cells as a replacement for stromal cells, with microfabrication to induce confinement.

Recent literature demonstrates that extracellular pH is a significant regulator of HSC differentiation and function [35]. Culturing HSCs at a slightly acidic pH of 6.9 (compared to the conventional neutral pH 7.4) maintains these cells in a less metabolically active and less proliferative state, thereby preserving their stemness and differentiation properties and enhancing their reconstitution potential following transplantation [13]. In our system, culturing HSCs within the 500‐µm deep microwells replicates well the formation of such acidic niches. It allows for the harnessing of pH as another modifiable factor to influence the fate and quality of HSCs in vitro.

Using this platform, we differentiated HSCs that express MHC‐II, CD11c, CD80, and CD86, typical antigen‐presenting markers and functional hallmarks of DCs, while retaining high CD34 expression, suggesting a yet immature state. This phenomenon is recognized in hematopoietic stem and progenitor cell (HSPC) biology, where differentiation does not always proceed synchronously for all markers, especially under in vitro conditions or nonphysiological stimulation [41]. The trends observed in Figures 3B and 6C appear, at first glance, to be inconsistent. In Figure 3B, marker upregulation increases with increasing well depth, whereas in Figure 6C the trend appears reversed, even in the cytokine‐only condition where no dsLBs are present. This apparent discrepancy is primarily due to differences in the control conditions used for normalization in the two datasets. In addition, variations in effective cell density between the experimental setups may contribute to the observed differences. These observations suggest that, in addition to acidification, other factors related to confinement and local cell density may also influence the differentiation response. From a therapeutic and applied perspective, retaining high CD34 expression in cells that otherwise display myeloid/DC markers could be advantageous in some contexts, as these cells may possess a greater capacity for engraftment and multilineage differentiation after transplantation—attributes linked with the immature HSPC state. However, truly functional DCs used for immunotherapies or vaccine platforms typically require full maturation with loss of CD34 (and CD14) and acquisition of professional antigen‐presenting capacity. Intermediate cells may thus be suboptimal for some DC‐based applications, but could still be valuable for regenerative approaches or as precursors adaptable to additional inputs [42].

Taken together, our results reflect a biologically plausible intermediate state that aligns with contemporary literature on HSPC plasticity, niche‐driven differentiation kinetics, and the challenges of pushing ex vivo differentiation to terminal DC fate. The relevance of these findings depends on the intended therapeutic use: for transplantation, preserving some progenitor properties may promote reconstitution; for immunotherapy, further maturation steps might be necessary.

Coculturing stem cells with feeder cells, such as fibroblasts, has long been a central strategy in HSC culture and differentiation [43]. In our study, we introduce synthetic cells as a replacement for conventional feeder layers, providing a more controllable, robust, and potentially safer platform for therapeutic applications. While our current system presents only a single ligand (DLL1), the use of NTA‐based coupling of recombinant ligands to the synthetic cell membrane enables straightforward expansion to a broad spectrum of other stroma‐associated molecules, such as vascular cell adhesion molecule 1 (VCAM1) or CXCL12. Unlike solid microparticle‐based systems used for artificial cell construction (e.g., Dynabeads for artificial antigen‐presenting cells), advanced synthetic cell models such as dsLBs more closely resemble key biophysical properties of stromal cells by mimicking their stiffness and lateral ligand mobility within the membrane. This results in a system that not only better replicates the natural stromal interface but also is compatible with upscaling and automation.

Cellular confinement represents another critical regulatory cue in HSC biology. Its effects range from promoting intercellular contacts (both homotypic and heterotypic), to restricting and guiding migration, altering local metabolite diffusion, turnover and access. Our system recapitulates several of these effects in a simplified manner by modulating metabolite diffusion through wells of varying depth and by inducing passive cellular jamming through physical entrapment. Our marker expression analyses collectively indicate that confinement significantly alters the resulting HSC phenotype as compared to a simple 2D culture, even under identical cytokine conditions. Although our current setup allows modulation of confinement through well depth, we cannot yet definitively disentangle the underlying mechanisms, whether driven by nutrient depletion, migratory restriction, or physical jamming effects.

Future developments of this system could not only expand the repertoire and combination of recombinant ligands displayed on synthetic cells but also incorporate additional components of the HSC niche, such as the extracellular matrix (ECM). Although not included in the current design, ECM components are known to critically regulate HSC differentiation through both biochemical and biophysical cues [16, 44]. Beyond contributing to cell confinement, ECM elements also serve as receptor ligands that can activate key signaling cascades, further refining the physiological relevance of synthetic HSC niche models. Overall, the presented system provides a versatile platform to integrate and independently assess biochemical and biophysical cues in HSC differentiation, enabled by synthetic cell engineering and microfabrication.

## 4 Materials and Methods

### 4.1 Cell Culture and Maintenance of HSCs

The murine bone marrow cell line FDCP‐Mix cl.A4 (RRID:CVCL_2040), hereafter called only FDCP cells (from Factor‐Dependent Continuous cell lines, Paterson laboratories), was purchased from the DSMZ‐German Collection of Microorganisms and Cell Cultures (Braunschweig, Germany). The cells were cultured in Iscove's Modified Dulbecco's Medium (IMDM) (Biowest, Nuaillé, France), containing L‐*Gln* and 25 mM (4‐(2‐hydroxyethyl)‐1‐piperazineethanesulfonic acid) (HEPES), supplemented with 20% Fetal Bovine Serum (FBS) (PAN‐Biotech, Aidenbach, Germany) and 1% Penicillin‐Streptomycin 100x Solution (VWR, Radnor, PA, USA) in a humidified incubator MCO‐50AIC (PHCbi, Tokyo, Japan) at 37°C/5% CO_2_. Sterile work was carried out in ScanLaf Mars class II biosafety cabinets (LaboGene, Lillerød, Denmark). Self‐renewal capacity was maintained through the addition of mouse IL‐3, 10 ng/ml (STEMCELL Technologies, Vancouver, Canada) every 2–3 days and passaged at around 1.5–2 × 10^6^/mL density in 25 cm^2^ polystyrene cell culture flasks (Greiner Bio‐One, Kremsmünster, Austria). Subculturing was carried out by centrifugation 6 min/900 rpm/room temperature (RT) using 5810 centrifuge (Eppendorf, Hamburg, Germany) and dilution down to 2.5 × 10^5^/mL. Cell counting was carried out on a CellDrop FLi (Wilmington, DE, USA). Backup cells were frozen in 70% IMDM, 20% FBS, and 10% dimethyl sulfoxide (DMSO) (Sigma–Aldrich, St. Louis, MO, USA) and stored at −80°C (FRYKA‐Kältetechnik, Esslingen am Neckar, Germany).

### 4.2 Generation of Small Unilamelar Vesicles (SUVs)

6 mM lipid solution was prepared with the following composition: 1% 1,2‐dioleoyl‐*sn*‐glycero‐3‐phosphoethanolamine labeled with Atto 488 (Atto 488 DOPE) (Sigma–Aldrich, St. Louis, MO, USA) and 20% L‐α‐phosphatidylglycerol (sodium salt) (Egg PG) were mixed with chicken egg L‐α‐phosphatidylcholine (Egg PC) (both from Avanti Research, Alabaster, AL, USA) up to 100 molar percent (all CHCl_3_ solutions) in a glass vial (neoLab, Heidelberg, Germany). As alternative fluorescent lipids in some experiments were utilized 1,2‐dioleoyl‐*sn*‐glycero‐3‐phosphoethanolamine‐N‐(lissamine rhodamine B sulfonyl) (ammonium salt) (Liss Rhod PE), 1,2‐dioleoyl‐*sn*‐glycero‐3‐phosphoethanolamine‐N‐(Cyanine 5) (18:1 Cy5 PE), or 1,2‐dioleoyl‐*sn*‐glycero‐3‐phosphoethanolamine‐N‐(carboxyfluorescein) (ammonium salt) (18:1 PE CF), always as 1% of total lipids. For experiments with protein coupling, 1,2‐dioleoyl‐*sn*‐glycero‐3‐[(N‐(5‐amino‐1‐carboxypentyl)iminodiacetic acid)succinyl] (nickel salt) (DGS‐NTA(Ni^2+^)) (all three from Avanti) was added up to 10%, adjusting down the Egg PC. The solvent was evaporated in a desiccator under vacuum for 20 min/RT. Phosphate‐buffered saline (PBS) (VWR, Radnor, PA, USA) was then added on top of the dried lipids and incubated 20 min/RT/dark. The swollen lipids were vortexed until completely dissolved, then extruded through a Whatman Nuclepore polycarbonate membrane with 100 nm pore size (Cytiva, Marlborough, MA, USA) using a Mini‐Extruder system by Avanti until the solution cleared, then stored at 4°C.

### 4.3 Synthetic Cell Assembly

Polydimethylsiloxane (PDMS) (SYLGARD 184 Silicone Elastomer Base) (Farnell, Leeds, UK) was collected with a 20‐mL disposable Omnifix syringe with Luer Lock (B. Braun, Melsungen, Germany). A 5‐µm Shirasu Porous Glas (SPG) filter for direct emulsification (SPG Technology, Miyazaki, Japan) was flushed with dH_2_O and attached to the syringe, which was then mounted on an AL‐1000 Aladdin SyringeONE Programmable Syringe Pump (World Precision Instruments, Sarasota, FL, USA). The filter was immersed into a DURAN borosilicate glass 3.3 beaker (DWK Life Sciences, Wertheim, Germany), filled with 400 mL 10 mM sodium dodecyl sulfate (SDS) (Merck, Darmstadt, Germany) solution, preheated to 65°C. 1 mL of the PDMS was emulsified with a speed of 325 μL/h in the SDS solution, moderately mixed on a magnetic stirrer C‐MAG HS 7 (IKA, Staufen im Breisgau, Germany). The resulting emulsion was collected in 50‐mL falcon tubes (Greiner Bio‐One, Kremsmünster, Austria) and centrifuged 5 min/300 g/RT on a Mega Star 4.0 R (VWR, Radnor, PA, USA). After discarding the supernatants, the pellets were pooled together and washed once with dH_2_O, after which the emulsion was stored in 5 mM SDS at RT.

1 mL emulsion (≈10^7^ droplets) was collected in an Eppendorf tube and pelleted 1 min/300 g/RT in a 5418 centrifuge (both from Eppendorf, Hamburg, Germany). The droplets were resuspended in 0.5 mL 1 mM SDS. MgCl_2_ (Sigma–Aldrich, St. Louis, MO, USA) was added to a concentration of 20 μM, quickly followed by 125 μL SUV solution of the corresponding type, mixed by hand and incubated 5 min/RT/dark. 1 mL Milli‐Q was added on top of the mix and the sample was centrifuged 2 min/300 g/RT. The resulting pellet was washed thrice with 1 mL 100 μM SDS with intermediate centrifugations of 1 min/300 g/RT. The droplets, now covered by membrane and from here on called dsLBs (from droplet‐supported Lipid Bilayer), were finally reconstituted with PBS and counted using the CellDrop system.

To analyze membrane formation on the dsLB surface, a µ‐Slide 8 Well chamber (ibidi, Gräfelfing, Germany) was washed twice with PBS and incubated with 5% Albumin fraction V (BSA) (Sigma–Aldrich, St. Louis, MO, USA) in PBS for 5 min/RT. The wells were washed twice with PBS; then, diluted dsLB samples were added and briefly left to settle down. Analysis was carried out with a BZ‐X810 inverted fluorescence phase contrast microscope, operated by BZ‐X800 Viewer and equipped with PlanFluor 10 × 0.3/14.5 mm Ph1 and PlanFluor 20 × 0.45/8.8–7.5 mm Ph1objectives; BZ‐X filter sets for green fluorescent protein (GFP) and Cy5, 40W high‐intensity light‐emitting diode (LED) fluorescence excitation lighting, and a 2/3 inch, 2.83 million pixel colorized monochrome charge‐coupled device (CCD) camera unit (all by Keyence, Osaka, Japan).

### 4.4 Protein Functionalization of Synthetic Cells

Synthetic cells were functionalized with the extracellular domain of mouse delta‐like protein 1 (mDLL1) through NTA‐based coupling, as follows: first, dsLBs were assembled with SUVs, containing 10% DGS‐NTA(Ni^2+^), as already described. A serial dilution of the same SUV sample (6 mM lipid concentration) with PBS was generated on a transparent flat‐bottomed 96‐well plate (Greiner Bio‐One, Kremsmünster, Austria), creating a concentration range of 0.5–60 μM. Fluorescence of the Atto 488 DOPE in the SUV/dsLB membrane was measured using Spark multimode microplate reader, operated by SparkControl v.3.2 software (both from Tecan, Männedorf, Switzerland), with in‐built gain optimization and excitation/emission settings adjusted to 485(±20)/535(±20)nm. Fluorescence‐based calibration curve was interpolated from the SUV dilutions using linear regression in Microsoft Excel (Microsoft, Redmond, WA, USA). From it, the total lipid concentration of the dsLB sample was extrapolated and thus the accessible DGS‐NTA molecules on the dsLB surface were calculated. Coupling of *His*‐tagged mDLL1 (Sino Biological, Beijing, China) was carried out in a ratio of one protein to four accessible NTA moieties. The reaction was executed in PBS, containing 2% BSA, in 0.5 mL protein LoBind tubes (Eppendorf, Hamburg, Germany) for 1 h/4°C/dark with occasional pipette mixing against sedimentation. The dsLBs were then washed twice with 2% BSA‐PBS, once with IMDM, with centrifugation steps of 1 min/300 g/RT, moving to a sterile environment, finally reconstituted with complete IMDM and counted using the CellDrop system. Coupled dsLBs were used in no more than 24h.

### 4.5 MESF Bead Assay

In order to quantify the amount of DLL1 that can be effectively presented on the dsLB surface, first the mDLL1‐*His* was labeled with the fluorophore Alexa Fluor 488 (AF488), as follows:

Recombinant mouse DLL1‐His‐Tag protein was reconstituted in sterile H_2_O per manufacturer's instructions and concentrated using a 3 kDa Amicon Ultra 0.5 mL Centrifugal Filter (Merck, Darmstadt, Germany). Buffer exchange was performed using a Bio‐Spin P‐6 Gel Column (Bio‐Rad Laboratories, Hercules, CA, USA) pre‐equilibrated with PBS. Protein concentration was measured at 0.7 mg/mL (NanoDrop One, ThermoFisher Scientific, Waltham, MA, USA). For fluorescent labeling, the protein was premixed with 1 M NaHCO_3_ buffer (pH 8.5) in 9/10 of the final reaction volume. Alexa Fluor 488 NHS‐Ester (ThermoFisher Scientific, Waltham, MA, USA) was diluted in DMSO at 20X molar excess in 1/10 of the final reaction volume and added to the protein. The reaction was incubated 1 h/RT. Unbound fluorophore was removed using a Bio‐Spin P‐6 Gel Column containing Tris Buffer. Final protein and dye concentrations were measured on the NanoDrop One, yielding a final protein‐to‐dye ratio of 1:7.5.

DsLBs were assembled, and their lipid concentration and from it the concentration of available DGS‐NTA(Ni^2+^) moieties were calculated as described in the previous section. The dsLBs were functionalized with the AF488‐labeled mDLL1‐*His*, as described above, but in multiple molar ratios of DLL1 to DGS‐NTA(Ni^2+^): 1:32, 1:16, 1:8, 1:4, 1:2, and 1:1. All samples were processed further as per the described protocol.

A cocktail of Quantum Alexa Fluor 488 MESF beads (Bangs Laboratories, Fishers, IN, USA) was prepared according to manufacturer's protocol. DsLBs or the bead cocktail were diluted 1:20 in 8‐well Nunc LabTeK glass bottom chamber slides (ThermoFisher Scientific, Waltham, MA, USA), filled with 200 μL PBS. Imaging was carried out on an LSM 880 Airyscan point laser scanning confocal microscope system, operated by ZEN Black 2.3 SP1 software, through a Plan‐Apochromat 20×/0.8 M27 objective, excited by 488 nm Ar laser, and filtered through 499–643 nm (all from Carl Zeiss, Oberkochen, Germany). The ImageJ (NIH, Bethesda, MD, USA) software was used for watershed particle separation, automated particle detection, and measurement of the mean fluorescent intensity of individual particles. The beads were used to generate a calibration curve to correlate fluorescent signal to MESF. The mean number of DLL1 molecules on the dsLB surface was calculated and used to generate another calibration curve linking the amounts of protein used to the protein density on the particles’ surface.

### 4.6 Fluorescence Recovery After Photobleaching (FRAP)

An 8‐well chamber for microscopy with Atto 488‐labeled dsLBs was prepared, as already described. Imaging was carried out with the LSM 880 confocal microscope, through a Plan‐Apochromat 63×/1.4 oil DIC M27 objective at 3× digital magnification, excited by 488 nm Ar laser, and filtered through 499–643 nm (all from Carl Zeiss, Oberkochen, Germany). Three regions of interest (ROIs) for each dataset were manually selected: ROI1 encompassed an entire dsLB, ROI2 selected an area of the dsLB membrane to be bleached, and ROI3 represented background. Data collection was carried out in the following sequence: five scans at 2% laser power, followed by a bleach scan of ROI2 at full power with a scan speed of 5 (8.19 µs/px), followed by 45 cycles every 2 s at max scan speed (13). Stack image data was analyzed using Fiji ImageJ (v1.54p) [45], reproducing the three ROIs. The data for all 30 cycles was uploaded to easyFRAP web‐based analysis tool (https://easyfrap.vmnet.upatras.gr/) [46] and FRAP was calculated. Results were normalized using either of the double or full scale options (checking the R^2^ fit). The data for mobile fraction, T‐half, and R^2^ were averaged from all image stacks.

### 4.7 Real‐Time Deformability Cytometry (RT‐DC)

DsLBs, generated as already described, were pelleted by centrifugation 1 min/300 g/RT and resuspended with a viscosity‐adjusted measurement buffer CellCarrier (Zellmechanik Dresden, Dresden, Germany), according to a Mcfarland standard of 6MFS. RT‐DC was performed on a setup comprised of an AcCellerator (Zellmechanik Dresden) and an Axiovert 200 M inverted microscope (Carl Zeiss, Oberkochen, Germany). The dsLBs were pushed through custom‐made PDMS microfluidics chips with 20 µm channels, generously provided by the lab of Fr. Lautenschläger (Saarland University, Saarbrücken, Germany), with flow rates of 80, 120, 140, 160, and 180 nL/s. The setup was operated through ShapeIn software (Zellmechanik Dresden) and imaging was recorded by a CMOS camera (Mikrotron, Unterschleiβheim, Germany). To analyze the recorded images in real time, enabling computation of a contour and projected area for each dsLB, the Shape‐Out 2 software (Zellmechanik Dresden) was used. To exclude dsLB doublets or debris area limits were set to 80–250 μm^2^ and deformation limits – to 0.003–0.008.

### 4.8 Generation of 3D Microenvironment for HSC Differentiation

Three square silicon wafers, 25 cm^2^ in size, were produced at the Institute of Semiconductors and Microsystems, Technical University of Dresden (Dresden, Germany). The wafers were covered with pillars sized either 100/200, 100/300, or 100/500 (width [µm]/height [µm]) in a square array. These were occasionally coated with (Tridecafluoro‐1,1,2,2‐tetrahydrooctyl)trichlorosilane, 97% (AB111444) (abcr, Karlsruhe, Germany) under vacuum to facilitate detachment of the cured PDMS. SYLGARD 184 Silicone Elastomer Base was mixed by hand with SYLGARD Silicone Elastomer Curing Agent (Farnell, Leeds, UK) in a ratio 12:1 (w/w), modified to achieve increased elasticity. After thorough homogenization it was poured on top of the wafers. Vacuum was applied via pump (VACUUBRAND, Wertheim, Germany) in a desiccator (Schott, Mainz, Germany) to suck out trapped air. The samples were left under vacuum until the bubbles disappeared, then transferred to a preheated oven UT 6060 (Heraeus, Hanau, Germany) and cured ON/60°C. The PDMS molds were then removed from the wafers by hand, creating an inert surface, containing microwells 100 µm in diameter and 200, 300, or 500 µm in depth respectively, and stored at RT. The microwell profile was validated on a confocal microscope using a 20× objective lens, operated by MahrSurf CM Explorer, and analyzed in MahrSurf MfM Extended v.9.2 (all from Mahr, Göttingen, Germany). For cell seeding, the molds were first cut into round pieces with a 14‐mm hollow punch (Boehm, La Fouillouse, France) and sealed to the bottom of wells of 24‐well plates (Greiner Bio‐One, Kremsmünster, Austria) using eco‐sil addition‐curing duplicating silicone for dental technology (Picodent, Wipperfürth, Germany) and cured at RT until solidification. The well plates were incubated 20min/RT/UV in a biosafety cabinet with 70% C_2_H_5_OH for sterilization, then washed with PBS under sterile conditions. The PBS was replaced with complete IMDM and the well‐plates were centrifuged 5 min/3000 rpm/RT and incubated ON/37°C/5% CO_2_ to facilitate medium entry into the microwells. Cells were seeded the following day.

### 4.9 Imaging of HSCs and Synthetic Cells in 3D Cocultures

A 24‐well plate with microwell plate pieces was prepared, as described in the previous section. A mix of FDCP cells (7.5 × 10^4^/mL) and dsLBs (3.75 × 10^4^/mL) in a ratio 2:1, supplemented with 10 ng/mL mIL‐3, was seeded on top of the microwells and cultured for 24 h at 37°C/5% CO_2_.

Light microscopy imaging was carried out on an Axiovert 5 RL TL SCB inverted microscope, equipped with LD A‐Plan 20×/0.35 Ph1, LD A‐Plan 40×/0.55 objectives, Axiocam 202 monochrome camera, and a 10 W white LED (all from Carl Zeiss, Oberkochen, Germany).

For fluorescence microscopy, the microwell samples were first washed twice with PBS, then fixed with 3% paraformaldehyde (PFA) (Thermo Fisher Scientific, Waltham, MA, USA) for 1 h/RT. After twice washing with PBS, the samples were stained with Hoechst 33342 (2.5 μM) and phalloidin‐AF647 (both from Thermo Fisher Scientific) for 1h/RT/dark. After washing out the stains with PBS the microwell pieces were extracted and placed upside down on the glass bottom of CELLView cell culture dishes (Greiner Bio‐One, Kremsmünster, Austria), maintaining humidity. Imaging was carried out with the LSM 880 confocal microscope through a Plan‐Apochromat 20×/0.8 M27 objective at 2× digital magnification. Data were recorded through individual excitation by 405‐nm diode, 488‐nm Ar, and 633‐nm HeNe lasers and collected through 411–502 nm, 499–607 nm, and 638–756 nm filter ranges respectively (all by Carl Zeiss, Oberkochen, Germany). Image composition was carried out with Fiji ImageJ.

### 4.10 Nanoindentation

A 24‐well plate with microwell plate pieces was prepared, as already described. Elasticity was measured at 37°C on a Pavone Nanoindenter, operated by Pavone Software v1.12, using a probe with stiffness 3.55 N/m, tip radius of 48.5 µm, and geo factor in air 2.97 (all from Optics11 Life, Amsterdam, The Netherlands). Poisson's ratio was set to 0.45. 1‐µm indentation was applied to the surface of the samples in between the microwells. Multiple measurements at different places of each sample were carried out and Young's modulus values were calculated directly within the instrument's operating software.

### 4.11 pH Analysis in 3D Cultures

A 24‐well plate with microwell plate pieces was prepared, as already described. FDCP cells were seeded at a concentration 7.5 × 10^4^/mL in each microwell size, as well as in control wells in 2D and cultured for 72 h at 37°C/5% CO_2_. On the day of measurement, 4‐µm nonfunctionalized silica microspheres (Bangs Laboratories, Fishers, IN, USA) were washed twice with PBS and pelleted via microcentrifugation (Thermo Fisher Scientific, Waltham, MA, USA). The beads were then resuspended with a premix of CF and Cy5 SUVs (25 µL each) (prepared as described previously) and incubated 10 min/RT/dark. The beads were washed twice again with PBS. For the generation of a standard curve, 1 mL of PBS samples, adjusted to pH 5.5, 6, 6.5, 7, 7.4, and 8, was added to the 24‐well plate next to the microwell cultures. The fluorescently labeled silica beads were added to each cell culture sample, as well as to the PBS wells, and incubated 30 min/37°C/5% CO_2_. Data acquisition was carried out on the LSM 880 confocal microscope through an EC Plan‐Neofluar 10×/0.3 M27 objective at 0.6× digital magnification, excited by 488‐nm Ar and 633‐nm HeNe lasers, with corresponding filters of 493–628 nm and 638–759 nm (all from Carl Zeiss, Oberkochen, Germany). For all samples, z‐stack images were collected over a manually selected depth with the recommended step size (≈15 µm). Data was extracted from maximal projections of the z‐stack images with Fiji ImageJ, followed by median filtering. ROIs were selected manually and mean gray value data were collected for each channel within the ROIs. For the standard curve, 50 ROIs were manually selected and measured. The data were exported to Microsoft Excel, where the CF intensity values were divided by the corresponding Cy5 values. A standard curve was built using the resulting ratios in a nonlinear regression (4PL) model. The ratiometric data from the various culture conditions were interpolated from the standard curve.

### 4.12 Alamar Blue Assay

A 24‐well plate with microwell plate pieces was prepared, as already described. In addition, PDMS was mixed with its elastomer curing agent in ratios 10:1, 12:1, and 14:1, poured and cured directly inside other wells of the 24‐well plate. FDCP cells were seeded at a concentration 10^5^/mL in each microwell size, on top of the flat‐surfaced PDMS, as well as in control wells in 2D and cultured for 48 h at 37°C/5% CO_2_, supplemented with 10 ng/mL mIL‐3.

For the Alamar Blue assay, 600 µL of the medium from each well was aspirated and wells were replenished with 400 µL complete IMDM. AlamarBlue viability reagent (Invitrogen, Carlsbad, CA, USA) was diluted 1:2 in complete IMDM and added at 100 µL/well (final 10% dye concentration), followed by incubation for 4 h/37°C/5% CO_2_. Supernatants (100 µL/well) were then transferred to a flat‐bottom 96‐well plate, and fluorescence was measured using Spark multimode microplate reader, with in‐built gain optimization and excitation/emission settings adjusted to 560/590 nm.

### 4.13 Analysis of Cell Cycle Progression

A 24‐well plate with microwell plate pieces was prepared, as already described. FDCP cells were seeded at a concentration 10^5^/mL in each microwell size, as well as in control wells in 2D, and cultured for 48 h at 37°C/5% CO_2_, supplemented with 10 ng/mL mIL‐3.

The FDCP cultures were extracted from the microwells. To facilitate that, the wells were washed with 5 mM ethylenediaminetetraacetic acid (EDTA) (Sigma–Aldrich, St. Louis, MO, USA) in PBS and incubated at 4°C. The microwell plate pieces were then dissociated from the well bottoms, turned upside down, squeezed with pincers, and were centrifuged 5 min/650 g/4°C.

The collected cells were transferred to a V‐bottom 96‐well plate for staining. Washing steps were carried out with PBS and centrifugation steps of 3 min/1500 rpm/4°C. After washing with PBS, the cells were fixed with ice‐cold 70% C_2_H_5_OH for 30 min/RT. The cells were further washed with PBS, then stained with FxCycle PI/RNase staining solution (Invitrogen, Carlsbad, CA, USA). After an incubation of exactly 25 min/RT/dark, the samples were directly measured by flow cytometry with excitation at 488 nm and emission collected by 590/40 nm filter.

### 4.14 Cytokine‐Induced HSC Differentiation in 2D Culture

FDCP cells were seeded in a 24‐well plate in a concentration 2.5 × 10^5^/mL for 10 days. Every second day, half of the medium was exchanged for fresh one, containing the corresponding cytokine treatment. In the control group, cells were maintained with mIL‐3, 7.5 ng/mL (a lower than standard concentration to reduce proliferation and baseline signaling). Treatment Regimen 1 consisted of mouse (m) GM‐CSF (PeproTech, Cranbury, NJ, USA) from day zero with the addition of mIL‐4 (Miltenyi Biotec, Bergisch Gladbach, Germany) from day four onwards, both at 500 U/mL. Treatment Regimen 2 consisted of mGM‐CSF, again from day zero every second day and one addition of mIL‐4 at day eight (again both at 500 U/mL). On day ten, the cells were harvested from the culture plate and analyzed by flow cytometry.

### 4.15 Cytokine‐Induced HSC Differentiation in 3D Culture

A 24‐well plate with microwell plate pieces was prepared, as already described. FDCP cells were seeded at a concentration 7.5 × 10^4^/mL in each microwell size and in neighboring wells without microwells for cytokines only treated samples. In the control group, cells were maintained with mIL‐3, 7.5 ng/mL, added every second day. For differentiation to dendritic cells, treatment Regimen 1 was used, described in the previous section. Medium was partially exchanged at these time points, depending on the state of consumption. The plate was cultured at 37°C/5% CO_2_. On day ten, the cells were harvested and analyzed by flow cytometry.

### 4.16 Synthetic Cell‐Induced HSC Differentiation

DsLBs were functionalized with mDLL1, as described previously and utilized to induce differentiation of the FDCP cells in combination with cytokines in 2D and 3D cultures. For 2D conditions, the cells (3 × 10^5^/mL) were mixed with dsLBs in a ratio ≈2:1 and cultured in a flat‐bottomed half area 96‐well plate (Greiner Bio‐One, Kremsmünster, Austria). For controls, the cells were also cocultured with nonfunctionalized dsLBs, or with cytokines alone.

For 3D cultures, first a 24‐well plate with microwell plate pieces was prepared, as already described. The FDCP cells (2.5 × 10^4^/mL) were mixed with DLL1‐functionalized dsLBs in ratio 1:1 and seeded on top of the microwells.

The cocultures (both 2D and 3D) were incubated at 37°C/5% CO_2_ for up to 10 days. Medium was partially exchanged in regular intervals, maintaining the cytokine regimen consistent with previous experiments. Nondifferentiated controls were maintained with mIL‐3 for viability reasons.

### 4.17 Antigen Loading

In the 3D differentiation assays, the FDCP cells were first extracted from the microwells, as already described. The collected cells were incubated with OVA peptide (323–339), labeled with fluorescein isothiocyanate (FITC) (Eurogentec, Seraing, Belgium) at concentration 1 µM in IMDM, containing 2% FBS, for 2 h/37°C/5% CO_2_ with occasional mixing. The cells were then washed twice with PBS to remove unbound peptide and transferred to a V‐bottomed 96‐well plate (Greiner Bio‐One, Kremsmünster, Austria) for further processing for flow cytometry.

### 4.18 Flow Cytometry

Cells were first stained with the viability marker Ghost Dye Red 780 (Cytek Biosciences, Fremont, CA, USA) (dil. 1000× in PBS) for 30 min/4°C/dark. Thereafter, all washing and incubation steps were carried out with FACS buffer (PBS, supplemented with 1% BSA, 2 mM EDTA and 0.02% NaN_3_ (Sigma–Aldrich, St. Louis, MO, USA)) and centrifugations of 3 min/1500 rpm/4°C. Next, the cells were incubated with mouse Fc block (α‐mCD16/CD32) (Cytek Biosciences, Fremont, CA, USA) (dil. 250×) for 20 min/4°C/dark. After a subsequent wash, the cells were incubated individually or in combination with the following fluorescently labeled antibodies: α‐mCD80‐Brilliant Violet (BV) 421, α‐mCD86‐BV605, α‐mCD34‐phycoerythrin (PE), α‐mNotch1‐allophycocyanin (APC), α‐mCD11c‐APC, and α‐mI‐A/I‐E‐AF700 (antibody against all I‐A and I‐E variants of the mouse MHC‐II molecules) (all from BioLegend, San Diego, CA, USA) (dil. 200x) for 20 min/4°C/dark. After a final wash, samples were measured on an Attune NxT flow cytometer (Thermo Fisher Scientific, Waltham, MA, USA), equipped with 405‐, 488‐, 561‐, and 637‐nm laser lines and a filter setup of 440/50 nm, 603/48 nm (violet), 530/30 nm (blue), 585/16 nm (yellow), 670/14 nm, 720/30 nm, and 780/60 nm (red). Compensation and data analysis were carried out on FlowJo v.10.3 (BD Biosciences, Franklin Lakes, NJ, USA). The following gating strategy was applied to all experimental data: first, living cells were gated, based on the Ghost Dye staining; then, doublets were excluded through plotting forward scatter area (FSC‐A) versus height (FSC‐H). Thereafter, for each cellular marker, where positive and negative populations were clearly distinguishable, the positive was collected as percentage of parent, or as mean fluorescence intensity (MFI). Where not, median fluorescence intensity (MdFI) of the whole singlets’ population was used.

### 4.19 Statistical Analysis and Data Representation

As this work has been performed with cells from the FDCP‐Mix cell line, no true biological replicates (i.e., donors) are possible. We therefore refer to biological replicates as those that were performed in independent experiments from different starting cultures. Technical replicates refer to parallel measurements performed in the same assays (mostly multiple wells on a well plate). Measurement replicates refer to individual but not independent measurements on the same sample (e.g., same PDMS mold). Data from individual experiments was normalized, thus converted into ratiometric form, then plotted together to demonstrate experimental reproducibility. All statistical analyses and graph generation were carried out in GraphPad Prism v.10.4.0 (GraphPad Software, San Diego, CA, USA). Mean values of biological or technical replicates ± standard deviation (SD) are represented in the graphs, unless specified otherwise. One‐way analysis of variance (ANOVA) test was utilized, unless specified otherwise. Statistical significance is illustrated as follows: *p < 0.05; **p < 0.01; ***p < 0.001; ****p < 0.0001; ns—nonsignificant. Other figures were generated using BioRender (https://www.biorender.com/) (BioRender, Toronto, Canada), Microsoft PowerPoint (Microsoft, Redmond, WA, USA), Adobe Illustrator 2019 (Adobe, Mountain View, CA, USA), Fiji ImageJ (v1.54p), MahrSurf MfM Extended v.9.2, and Prova (Optics11 Life, Amsterdam, The Netherlands).

## Supporting Information

Additional supporting information can be found online in the Supporting Information section.

## Funding

This study was supported by Deutsche Forschungsgemeinschaft (525255627, 545610076), Joachim Herz Stiftung, Daimler und Benz Stiftung (32‐12/22).

## Conflicts of Interest

The authors declare no conflicts of interest.

## Supporting information

Supplementary Material

## Data Availability

The data that support the findings of this study are available from the corresponding author upon reasonable request.
