# Supplementary material for: Synthetic Cell‐Based Artificial Stem Cell Niches for Hematopoietic Stem Cell Differentiation
Source: Chembiochem. 2026 Apr 21;27(8):e202500864. doi: 10.1002/cbic.202500864 (PMC13096856; doi:10.1002/cbic.202500864)
Supplement: Supplementary file 1 — Supplementary Material [file CBIC-27-e202500864-s001.pdf]

Supporting information

Synthetic Cell-based Artificial Stem Cell Niches for Hematopoietic Stem Cell Differentiation

Authors: Ivaylo Balabanov[1], Sara Madureira[1], Anna Burgstaller[1,2], Maja Fehlberg[1], Nils Piernitzki[1,2], Nurzhan Abdukarimov[1], Franziska Lautenschläger[3,4,5], Oskar Staufer[1,2,3,4,6]

[1]INM - Leibniz Institute for New Materials, Campus D2 2, 66123 Saarbrücken, Germany

[2]Helmholtz Institute for Pharmaceutical Research Saarland, Helmholtz Center for Infection Research, Campus E8 1, 66123 Saarbrücken, Germany

[3]Center for Biophysics, Saarland University, Campus Saarland, 66123 Saarbrücken, Germany

[4]Max Planck School Matter to Life, Jahnstraße 29, 66120 Heidelberg, Germany

[5]NT Faculty, Experimental Physics, Saarland University

[6]Max Planck Bristol Centre for Minimal Biology, Cantock's Close, Bristol BS8 1TS, United Kingdom

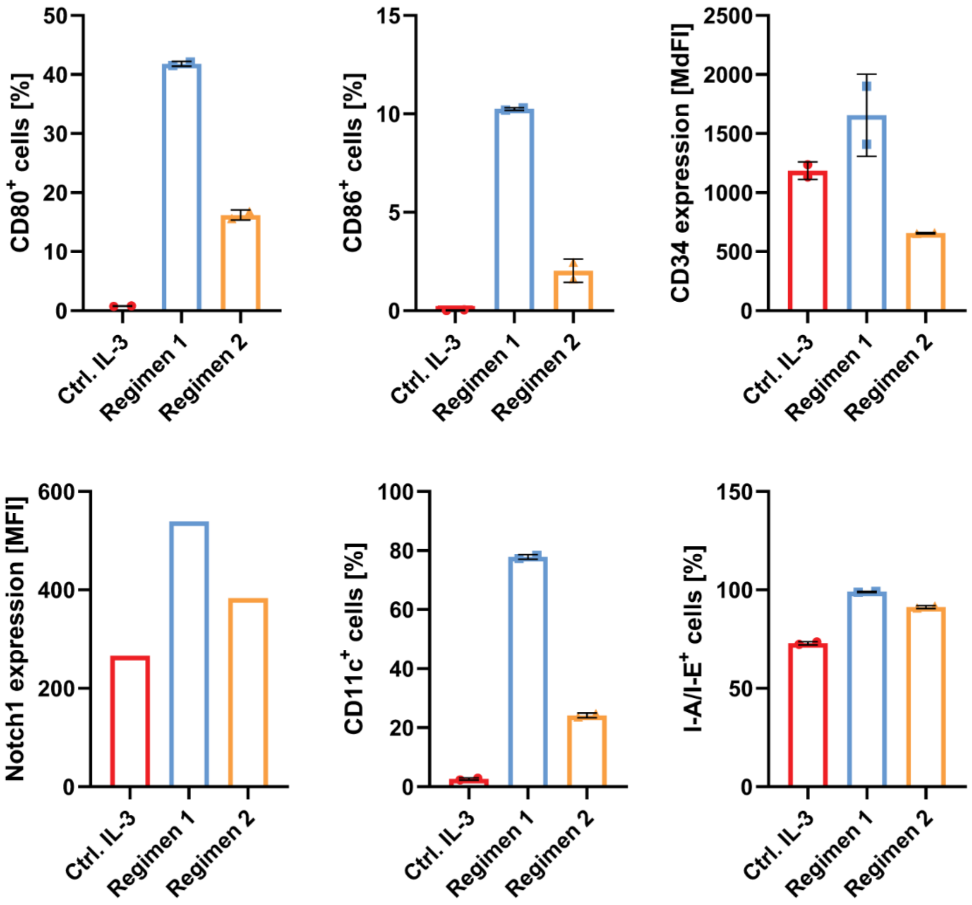

Supplementary Figure 1: HSC differentiation in 2D. *Flow cytometric analysis of FDCP-Mix cells after 10-day differentiation in 2D culture, induced by administration of GM-CSF and IL-4 in two distinct regimens. For control cells were maintained in IL-3-supplemented medium as a necessary survival signal. Data is represented as either positive population of singlets, median fluorescence intensity (MdFI) or mean fluorescence intensity (MFI) of singlets, or as MFI of positive population. Results are shown as mean ± SD from n=2 technical replicates from one of two independent experiments.*

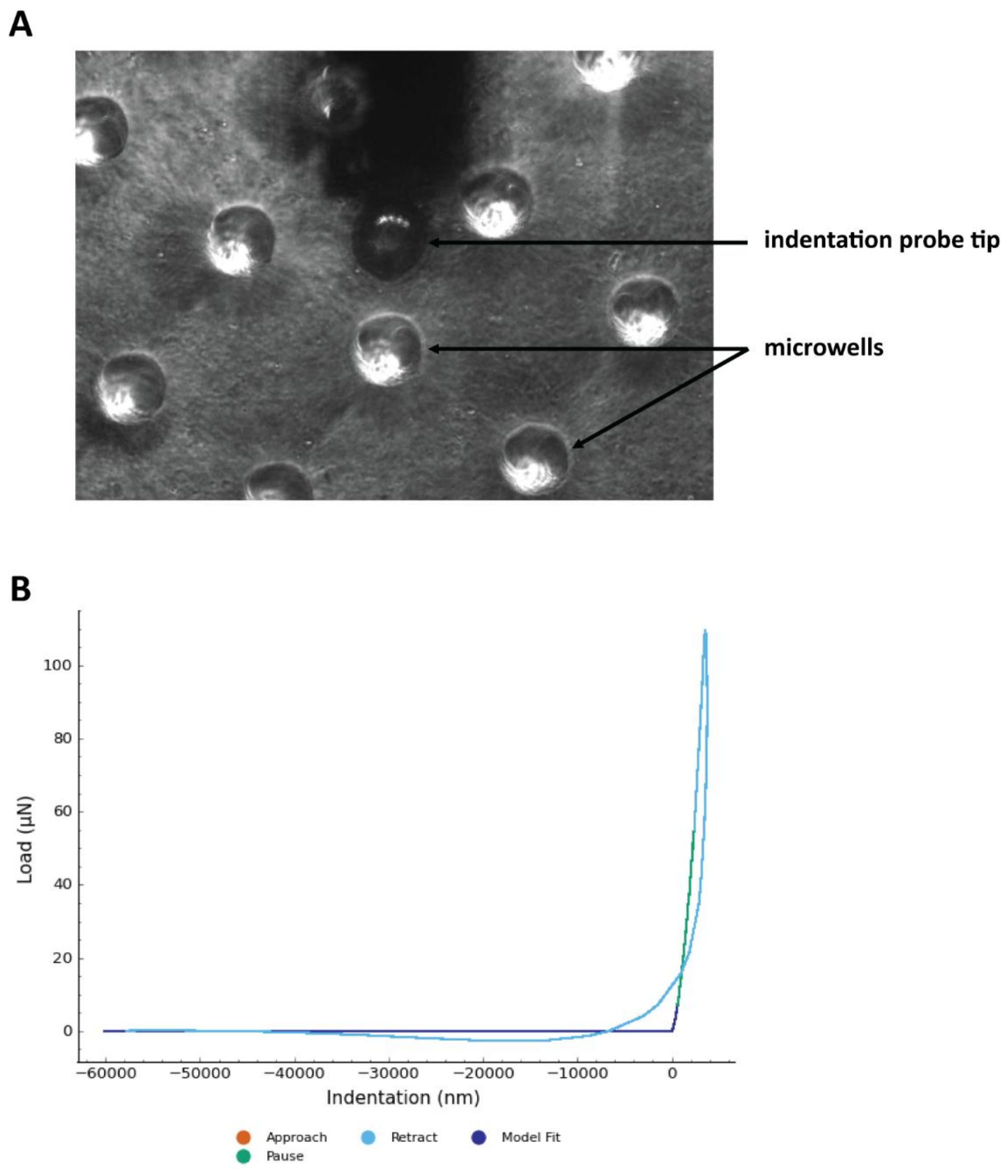

Supplementary Figure 2: Nanoindentation analysis of microwell-containing PDMS substrate. (A) *Representative micrograph acquired during indentation measurement. The tip of the cantilever force probe indicates the position of contact. These were chosen to be equidistant from the nearby microwells.* ***(B)*** *Representative load–indentation curve recorded on the sample surface. Load (µN) is plotted versus indenter displacement (nm), with negative indentation values indicating the indenter moving into the surface. The orange trace shows the approach segment as the indenter is driven into the material until the programmed maximum load is reached. The green trace marks the brief pause/hold period at maximum load, during which the indenter position is maintained to monitor time-dependent viscoelastic behavior. The light-blue trace corresponds to the retract segment as the load is removed and the*

indenter is withdrawn, revealing elastic recovery and any residual indentation depth. The dark-blue line denotes the model fit to the unloading portion of the curve, which is used by the nanoindenter software to calculate the elastic modulus.

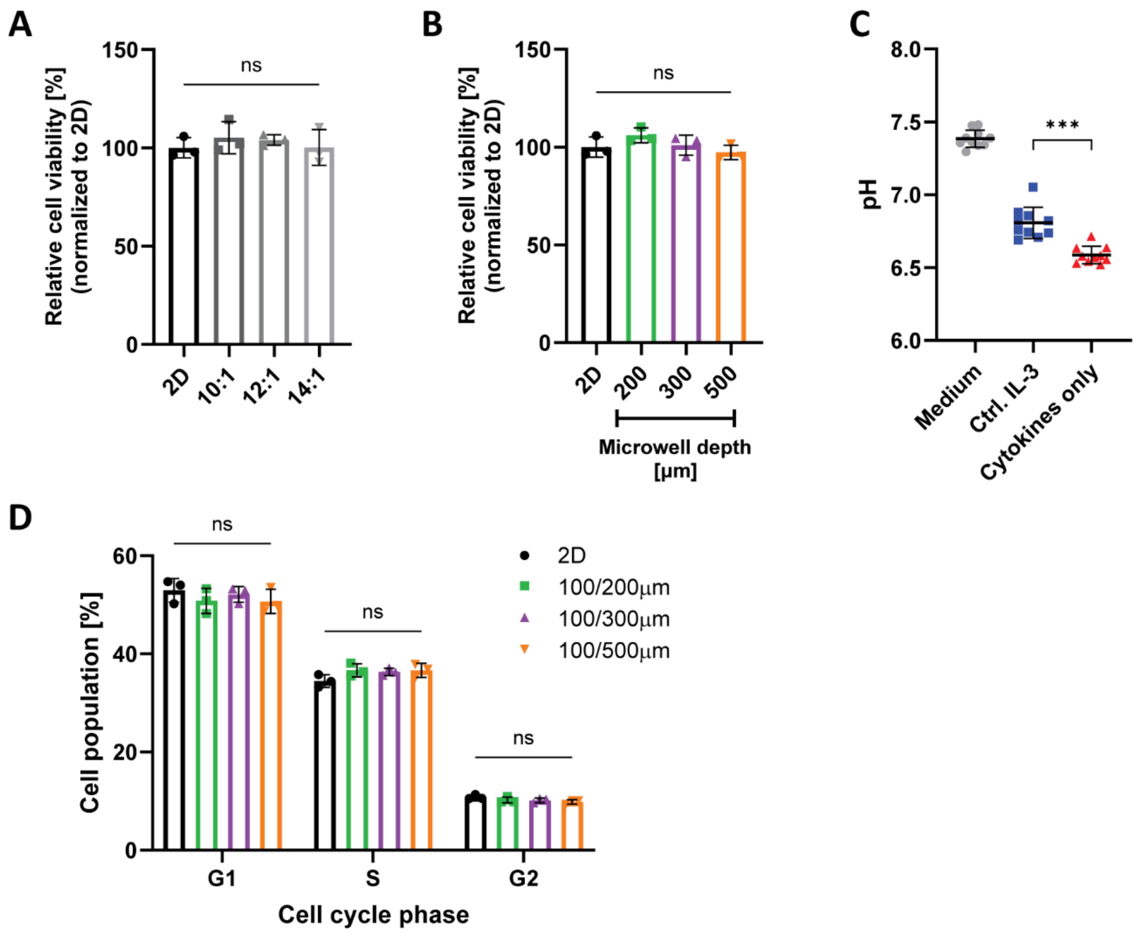

Supplementary Figure 3: Biocompatibility of PDMS substrates of different characteristics for HSC growth. *Comparison of viability and metabolic activity of FDCP-Mix cells, cultured **(A)** on top of flat-surfaces PDMS, cured at different ratios to the crosslinking agent, or **(B)** inside microwells of increasing depth, all in comparison to cells, grown on standard cell culture treated polystyrene for 48h and assessed by Alamar blue assay. The results demonstrate the inertness of PDMS towards the cell line and also that the microwell depth does not affect cell growth. Data shown as mean ± SD from n=3 technical replicates. Statistical analysis was performed with one-way ANOVA, combined with Dunnett's multiple comparisons test with a single pooled variance. **(C)** Fluorescence-based ratiometric pH-sensing measurements, performed after eight days of culture of FDCP-Mix cells with either control (blue) or differentiating (red) cytokines. Measurements of the culture medium (grey) show the method's accuracy. Statistical analysis was performed with Brown-Forsythe and Welch ANOVA tests, combined with Dunnett's T3 multiple comparisons test with individual variances computed for each comparison. **(D)** Comparison of proliferation of FDCP-Mix cells, cultured in the same experimental conditions, as in (B) shows no effect of microwell depth on proliferation. Analysis of cell cycle progression was performed via staining with*

*propidium iodide (PI) and measured by flow cytometry. Statistical analysis was performed with repeated measures two-way ANOVA with Geisser-Greenhouse correction, combined with Dunnett's T3 multiple comparisons test with individual variances computed for each comparison. Symbols in all graphs represent *p<0,05; **p<0,01; ***p<0,001; ****p<0,0001 and ns as non-significant.*

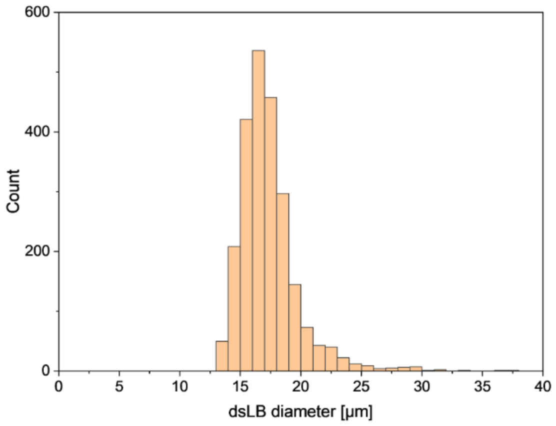

Supplementary Figure 4: Size distribution analysis of the emulsified PDMS-based synthetic cells, generated for this study. *Confocal microscopy images were analyzed by ImageJ for particle size, using automatic particle detection algorithm. Results show dsLBs have homogenous size profile, comparable with in vitro cell cultures.*
